# Novel predictors of neurosyphilis among HIV-negative syphilis patients with neurological symptoms: an observational study

**DOI:** 10.1186/s12879-017-2339-3

**Published:** 2017-04-26

**Authors:** Yao Xiao, Man-Li Tong, Li-Li Liu, Li-Rong Lin, Mei-Jun Chen, Hui-Lin Zhang, Wei-Hong Zheng, Shu-Lian Li, Hui-Ling Lin, Zhi-Feng Lin, Hui-Qin Xing, Jian-Jun Niu, Tian-Ci Yang

**Affiliations:** 1 Zhongshan Hospital, Medical College of Xiamen University, Xiamen, 361004 China; 2 Xiamen Hospital of Traditional Chinese Medicine, Xiamen, 361009 China; 3 Xiamen Zhongshan Hospital, Fujian Medical University, Xiamen, 361004 China; 4 Xiamen Huli District Maternity and Child Care Hospital, Xiamen, 361009 China; 5 Institute of Neuroscience, Medical College of Xiamen University, Xiamen, 361005 China

**Keywords:** Symptomatic neurosyphilis, Predictors, Lumbar puncture, Rapid plasma reagin, *Treponema pallidum* particle agglutination

## Abstract

**Background:**

Known predictors of neurosyphilis were mainly drawn from human immunodeficiency virus (HIV)-infected syphilis patients, which may not be applicable to HIV-negative populations as they have different characteristics, particularly those with neurological symptoms. This study aimed to identify novel predictors of HIV-negative symptomatic neurosyphilis (S-NS).

**Methods:**

From June 2005 to June 2015, 370 HIV-negative syphilis patients with neurological symptoms were recruited, consisting of 191 S-NS patients (including 123 confirmed neurosyphilis and 68 probable neurosyphilis patients) and 179 syphilis/non-neurosyphilis (N-NS) patients. Clinical and laboratory characteristics of S-NS were compared with N-NS to identify factors predictive of S-NS. Serum rapid plasma reagin (RPR), *Treponema pallidum* particle agglutination (TPPA), and their parallel testing format for screening S-NS were evaluated.

**Results:**

The likelihood of S-NS was positively associated with the serum RPR and TPPA titers. The serum TPPA titers performed better than the serum RPR titers in screening S-NS. The optimal cut-off points to recognize S-NS were serum RPR titer ≥1:4 and serum TPPA titer ≥1:2560 respectively. A parallel testing format of a serum RPR titer ≥1:2 and serum TPPA titer ≥1:1280 screened out 95.8% of S-NS and all confirmed cases of neurosyphilis. S-NS was independently associated with male sex, serum RPR titer ≥1:4, serum TPPA titer ≥1:2560, and elevated serum creatine kinase. Concurrence of these factors increased the likelihood of S-NS.

**Conclusions:**

Quantitation of serum TPPA is worthwhile and performs better than serum RPR in screening S-NS. Serum RPR, serum TPPA, male sex, and serum creatine kinase can predict S-NS. Moreover, patients with both a serum RPR titer <1:2 and a serum TPPA titer <1:1280 have a low probability of S-NS, suggesting that it is reasonable to reduce lumbar punctures in such individuals.

## Background

Syphilis, caused by *Treponema pallidum*, remains the third prevalent notifiable infectious disease in China in the past decade (http://www.stats.gov.cn/english/). Neurosyphilis can develop at any time in the course of syphilis. The organism invades the central nervous system in the early course of disease and likely disseminates before the clinical manifestations of primary syphilis, which is called “neuroinvasion” [1]. Those who fail to clear the organisms are deemed to have “asymptomatic neurosyphilis” and are at risk for subsequent development of neurological symptoms. The clinical manifestations of symptomatic neurosyphilis (S-NS) are protean, exact replicas of the symptoms of various neurological disorders, and it is difficult to distinguish S-NS from other central nervous system diseases, which can significantly delay treatment. Conventionally, diagnosis of neurosyphilis is based on a reactive venereal disease research laboratory (VDRL) test, pleocytosis and/or elevated protein concentrations in cerebrospinal fluid (CSF) collected by lumbar puncture. Although lumbar puncture is invasive, syphilis patients would be considered for CSF assessment on the condition of clinical evidence of neurological, ocular or auricular involvement; tertiary syphilis; congenital syphilis; or failure to achieve an adequate response to treatment [2, 3]. Some experts even recommend CSF examination in all human immunodeficiency virus (HIV)-infected individuals with any stage of syphilis [3]. Researchers have sought to identify predictors for the early recognition of neurosyphilis to avoid unnecessary lumbar puncture. Recently, neurosyphilis was found to be more prevalent in HIV-infected individuals with high (≥1:32) serum rapid plasma reagin (RPR) titers or low (<350 cells/μL) peripheral blood CD4+ T cell counts, and lumbar puncture was recommended in such individuals [4]. However, HIV-negative individuals seemed to have lower serum VDRL and *T. pallidum* particle agglutination (TPPA) titers [5]; therefore, such a selective approach for lumbar puncture may not be applicable to the scenario where syphilis is prevalent among HIV-negative key populations, such as China [6]. A survey showed 13.6% of HIV-negative syphilis patients were diagnosed with S-NS [7].

To the best of our knowledge, previous studies have rarely concentrated on HIV-negative syphilis patients [8–10], particularly those with neurological symptoms. This study aimed to identify predictive factors of S-NS and to explore serological indicators for lumbar puncture in HIV-negative syphilis patients with neurological symptoms over a ten-year period.

## Methods

### Study population and ethics statement

**Fig. 1:**
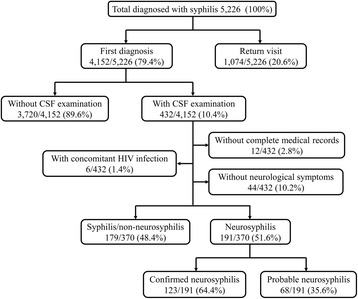
The flow of participants from enrollment to analysis

This study was conducted at Zhongshan Hospital, Medical College of Xiamen University from June 2005 to June 2015. A total of 5,226 hospitalized patients were clinically diagnosed with syphilis by combining serodiagnosis and medical history. Patients were excluded from the study as follows: return visit, without lumbar puncture, without complete medical records, without neurological symptoms, HIV infection. At last, 370 HIV-negative syphilis patients with neurological symptoms were enrolled in this study (Fig. 1). Clinical information was extracted and recorded for each participant, including medical history, physical examination and laboratory investigation. This study was approved by the Institutional Ethics Committee of Zhongshan Hospital, Medical College of Xiamen University, and it was in compliance with national legislation and the Declaration of Helsinki guidelines. Written informed consent was obtained from all study participants.

### Diagnostic criteria

The diagnostic criteria for neurosyphilis complied with the guidelines of the Centers for Disease Control in America and Europe [2, 11]. The neurosyphilis cases were subdivided into confirmed neurosyphilis and probable neurosyphilis. Confirmed neurosyphilis is defined as syphilis of any stage with reactive RPR in CSF. Probable neurosyphilis is defined as syphilis of any stage with a negative CSF-RPR test and both of the following: (1) elevated CSF protein (>500 mg/L) and/or leukocyte count (>10 cells/μL) in the absence of other known causes of these abnormalities and (2) clinical symptoms or signs consistent with neurosyphilis without other known causes for these clinical abnormalities. Syphilis/non-neurosyphilis (N-NS) was defined as syphilis of any stage excluding neurosyphilis.

### Laboratory tests

The syphilitic serological tests for each sample were performed using RPR (InTec, Xiamen, China) and TPPA (Fujirebio, Tokyo, Japan) tests according to the manufacturer's instructions and as previously reported [12]. Other biochemical parameters were also consistent with our previous studies [12–14], including CSF protein, albumin, lactate, lactate dehydrogenase, chloride, glucose, and leukocyte count, as well as blood HbA1c, fasting plasma glucoses, creatine kinase (CK), homocysteine, triglyceride, total cholesterol, alanine transaminase, aspartate transaminase, total bilirubin, urea nitrogen and creatinine.

### Statistical analysis

The Mann-Whitney U test was used for continuous variables with skewed distribution, and a *χ* ^2^ test or Fisher’s exact test was used for categorical variables. The logistic regression model was used to test the associations between different variables and the development of neurosyphilis. Spearman's rank correlation was used to analyze the correlation between serum RPR and TPPA reactivity. The above-mentioned statistical analyses were performed using SPSS 19.0 for Windows (SPSS Inc., Chicago, Illinois, USA). Receiver operating characteristic (ROC) analysis was performed to determine the performance of serum RPR and TPPA titer in screening S-NS, and the optimal cut-off points were determined corresponding to the maximal Youden’s index (sensitivity+specificity−100%). Comparison of ROC curves was carried out using Medcalc version 12.3.0 (Broekstraat 52, 9030; Mariakerke, Belgium). A two-sided *P*-value of <0.05 was considered statistically significant.

## Results

### Characteristics of the study participants

Over a ten-year period, a total of 370 HIV-negative syphilis patients with neurological symptoms were included in this study, consisting of 191 S-NS and 179 N-NS patients (Fig. 1). One hundred and twenty-three of the 191 S-NS patients met the definition of confirmed neurosyphilis, and 68 were defined as probable neurosyphilis. Overall, 70.5% (261/370) of these participants were males, accounting for 78.0% in the S-NS group and 62.6% in the N-NS group (*P*=0.001). The median age of all study participants was 53 (range, 21-85). There was no significant difference in age distribution between S-NS and N-NS patients (*P*=0.661). The most common neurological symptom of S-NS patients was cognitive decline (31.4%), followed by hemiparesis or hemiplegia (17.3%), dysbasia (16.8%). Other less common symptoms (<15%) included: aphasia, seizure, headache, disturbance of consciousness, bowel and bladder dysfunction, vision loss, vomiting, vertigo, neck stiffness, numbness in the lower extremities. S-NS patients had significantly higher serum RPR titers (median, 1:16; interquartile range [IQR], 1:4-1:64) and serum TPPA titers (median, 1:10240; IQR, 1:2560-1:20480) than N-NS patients (*P*<0.001). CSF protein, albumin and lactate levels and CSF leukocyte counts were higher and CSF glucose lower in the S-NS group than in the N-NS group (*P*<0.05). There was no difference between S-NS and N-NS in intracranial pressure, CSF chloride and lactate dehydrogenase. Among the 191 S-NS patients, those with probable neurosyphilis had lower serum RPR titers (median, 1:2 versus 1:32, *P*<0.001) and serum TPPA titers (median, 1:2560 versus 1:20480, *P*<0.001) than patients with confirmed neurosyphilis. CSF RPR and TPPA titers, CSF leukocyte counts and protein levels were also lower, whereas CSF glucose and lactate dehydrogenase were higher in the probable neurosyphilis subgroup than in the confirmed neurosyphilis subgroup (*P*<0.05) (Table 1).

**Table 1:** Clinical characteristics of the study participants

	N-NS (n=179)	S-NS (n=191)	*P* ^a^	S-NS		*P* ^*b*^	*P* ^*c*^	*P* ^*d*^
				Confirmed (*n*=123)	Probable (*n*=68)			
Male	112 (62.6%)	149 (78.0%)	**0.001**	97 (78.9%)	52 (76.5%)	0.702	**0.003**	**0.039**
	P_50_ (P_25_-P_75_)^e^	P_50_ (P_25_-P_75_)		P_50_ (P_25_-P_75_)	P_50_ (P_25_-P_75_)			
Age	54 (45-64)	53 (46-61)	0.661	52 (46-60)	55 (45-64)	0.373	0.474	0.869
Serum RPR titer	1:1 (Negative-1:2)	1:16 (1:4-1:64)	**<0.001**	1:32 (1:16-1:64)	1:2 (1:1-1:8)	**<0.001**	**<0.001**	**<0.001**
Serum TPPA titer	1:640 (1:320-1:1280)	1:10240 (1:2560-1:20480)	**<0.001**	1:20480 (1:10240-1:20480)	1:2560 (1:1280-1:5120)	**<0.001**	**<0.001**	**<0.001**
CSF RPR titer	Negative	1:2 (Negative-1:4)	**<0.001**	1:4 (1:2-1:8)	Negative	**<0.001**	**<0.001**	1.0
CSF TPPA titer	Negative	1:1280 (1:160-1:5120)	**<0.001**	1:5120 (1:1280-1:10240)	1:80 (1:80-1:640)	**<0.001**	**<0.001**	**<0.001**
CSF leukocyte (cells/μL)	4 (2-7)	15 (6-48)	**<0.001**	29 (12-59)	6 (2-13)	**<0.001**	**<0.001**	**0.015**
CSF protein (mg/L)	390.9 (289.9-540.0)	606.0 (418.6-884.3)	**<0.001**	733.9 (502.0-1025.0)	443.1 (325.8-607.2)	**<0.001**	**<0.001**	0.183
CSF albumin (mg/L)	249.0 (173.7-361.9)	296.4 (220.0-456.2)	**0.003**	296.4 (243.0-500.6)	297.0 (182.7-446.2)	0.218	**0.001**	0.204
CSF glucose (mmol/L)	3.70 (3.40-4.20)	3.54 (3.21-4.19)	**0.037**	3.48 (3.12-4.08)	3.74 (3.25-4.20)	**0.030**	**0.003**	0.913
CSF chloride (mmol/L)	124.3 (121.9-126.4)	124.4 (121.5-126.3)	0.696	124.8 (121.5-126.5)	123.9 (121.6-125.9)	0.303	0.902	0.330
CSF lactate (mmol/L)	1.76 (1.57-2.02)	1.89 (1.67-2.17)	**0.043**	1.90 (1.73-2.30)	1.83 (1.66-2.06)	0.160	**0.018**	0.516
CSF lactate dehydrogenase (U/L)	20.2 (16.7-27.7)	20.2 (16.0-26.5)	0.696	19.5 (16.0-23.6)	22.2 (17.8-34.1)	**0.032**	0.131	0.184
Intracranial pressure (mmH_2_O)	135 (110-160)	140 (110-160)	0.604	140 (110-160)	140 (100-160)	0.882	0.656	0.692

*N-NS* syphilis/non-neurosyphilis, *S-NS* symptomatic neurosyphilis, *RPR* rapid plasma reagin, *TPPA T. pallidum* particle agglutination, *CSF* cerebrospinal fluid, *P* ^*a*^ Comparison between the S-NS group and the N-NS group, *P* ^*b*^ Comparison between the confirmed neurosyphilis subgroup and the probable neurosyphilis subgroup, *P* ^*c*^ Comparison between the confirmed neurosyphilis subgroup and the N-NS, *P* ^*d*^ Comparison between the probable neurosyphilis subgroup and the N-NS

^e^Data with skewed distribution was described using median (interquartile range)

### Serum RPR and serum TPPA reactivity associated with S-NS

**Fig. 2:**
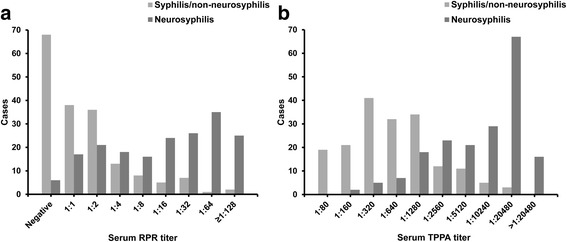
Serological reactions to syphilis among S-NS and N-NS patients: **(a)** Serum RPR reactivity; **(b)** Serum TPPA reactivity. Units on the Y axis described the number of participants whose results was in each bar

We compared the serological characteristics between S-NS and N-NS. As the serum RPR titer increased, the number of S-NS cases gradually increased (Fig. 2a). The association with S-NS increased significantly with the serum RPR titers (*P* for trend <0.001) (Table 2). Patients with higher serum RPR titers compared to patients with relatively lower serum RPR titers (e.g., serum RPR titer ≥1:2 versus serum RPR titer <1:2) were more likely to be diagnosed with S-NS. Similarly, as the serum TPPA titer increased, the number of S-NS cases increased, and a clustered peak of values can be observed to the right in Fig. 2b. The likelihood of S-NS was positively associated with the reactivity of serum TPPA (*P* for trend <0.001) (Table 2). Patients with higher serum TPPA titers compared to patients with relatively lower serum TPPA titers (e.g., serum TPPA titer ≥1:1280 versus serum TPPA titer <1:1280) were more likely to be diagnosed with S-NS.

**Table 2:** Serum RPR and serum TPPA reactivity associated with S-NS

	S-NS n (%)	N-NS n (%)	OR	*P*		S-NS n (%)	N-NS n (%)	OR	*P*
Serum RPR titers					Serum RPR titers				
Negative	6(3.2)	68(38.2)	1.0 (Ref.)						
1:1	17(9.0)	38(21.3)	5.070	**0.002**	≥1:1 (vs <1:1)	182(96.8)	110(61.8)	18.752	**<0.001**
1:2	21(11.2)	36(20.2)	6.611	**<0.001**	≥1:2 (vs <1:2)	165(87.8)	72(40.4)	10.562	**<0.001**
1:4	18(9.6)	13(7.3)	15.692	**<0.001**	≥1:4 (vs <1:4)	144(76.6)	36(20.2)	12.909	**<0.001**
1:8	16(8.5)	8(4.5)	22.667	**<0.001**	≥1:8 (vs <1:8)	126(67.0)	23(12.9)	13.696	**<0.001**
1:16	24(12.8)	5(2.8)	54.400	**<0.001**	≥1:16 (vs <1:16)	110(58.5)	15(8.4)	15.325	**<0.001**
1:32	26(13.8)	7(3.9)	42.095	**<0.001**	≥1:32 (vs <1:32)	86(45.7)	10(5.6)	14.165	**<0.001**
1:64	35(18.6)	1(0.6)	396.667	**<0.001**	≥1:64 (vs <1:64)	60(31.9)	3(1.7)	27.344	**<0.001**
≥1:128	25(13.3)	2(1.1)	141.667^†^	**<0.001**	≥1:128 (vs <1:128)	25(13.3)	2(1.1)	13.497	**<0.001**
**Serum TPPA titers**					**Serum TPPA titers**				
≤1:160^a^	2(1.1)	40(22.5)	1.0 (Ref.)						
1:320	5(2.7)	41(23.0)	2.439	0.303	≥1:320 (vs <1:320)	186(98.9)	138(77.5)	26.957	**<0.001**
1:640	7(3.7)	32(18.0)	4.375	0.078	≥1:640 (vs <1:640)	181(96.3)	97(54.5)	21.592	**<0.001**
1:1280	18(9.6)	34(19.1)	10.588	**0.003**	≥1:1280 (vs <1:1280)	174(92.6)	65(36.5)	21.607	**<0.001**
1:2560	23(12.2)	12(6.7)	38.333	**<0.001**	≥1:2560 (vs <1:2560)	156(83.0)	31(17.4)	23.117	**<0.001**
1:5120	21(11.2)	11(6.2)	38.182	**<0.001**	≥1:5120 (vs <1:5120)	133(70.7)	19(10.7)	20.236	**<0.001**
1:10240	29(15.4)	5(2.8)	116.000	**<0.001**	≥1:10240 (vs <1:10240)	112(59.6)	8(4.5)	31.316	**<0.001**
≥1:20480	83(44.1)	3(1.7)	553.333^‡^	**<0.001**	≥1:20480 (vs <1:20480)	83(44.1)	3(1.7)	46.111	**<0.001**

*S-NS* symptomatic neurosyphilis, *N-NS* syphilis/non-neurosyphilis, *RPR* rapid plasma reagin, *TPPA T. pallidum* particle agglutination, *OR* odds ratio, *Ref*., reference

^a^This group included two S-NS patients and 21 N-NS patients with serum TPPA titers of 1:160 and 19 N-NS patients with serum TPPA titers of 1:80

^†^ *P* for trend <0.001

^‡^ *P* for trend <0.001

### Screening performance of serum RPR and serum TPPA for S-NS

**Fig. 3:**
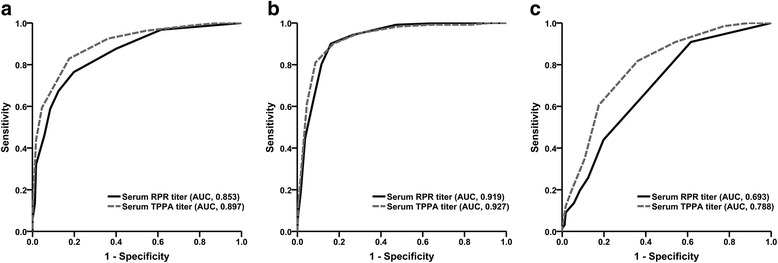
Screening performance of serum RPR and serum TPPA reactivity: **(a)** for the identification of S-NS; **(b)** for the identification of confirmed neurosyphilis; **(c)** for the identification of probable neurosyphilis

Using the serological tests for syphilis as screening tests for S-NS, the screening accuracy of serum RPR and serum TPPA were reflected using the ROC curve and area under the curve (AUC). The AUC for serum RPR titer was 0.853 (95% CI, 0.813 to 0.888). Surprisingly, the performance of serum TPPA titer was significantly better than that of serum RPR titer (AUC, 0.897; 95% CI, 0.861 to 0.926; *P*=0.006) (Fig. 3a). We also evaluated the accuracy in screening confirmed neurosyphilis (i.e., reactive CSF-RPR). For the identification of confirmed neurosyphilis, the AUC for serum TPPA titer reached up to 0.927, similar to that for serum RPR titer (AUC, 0.919; *P*=0.622) (Fig. 3b). Serum RPR and serum TPPA tests were slightly inferior for screening probable neurosyphilis, but serum TPPA titer still had a relatively better performance than serum RPR titer (*P*=0.003) (Fig. 3c). Furthermore, we also found a correlation between serum RPR and serum TPPA reactivity (*r*_*s*_=0.775, *P*<0.001). The optimal cut-off points of serological tests for the identification of S-NS were serum RPR titer ≥1:4 and serum TPPA titer ≥1:2560 (Fig. 3a). A threshold of serum RPR titer ≥1:4 provided a sensitivity of 76.6%, specificity of 79.8% and negative predictive values (NPV) of 76.3% in screening S-NS. A threshold of serum TPPA titer ≥1:2560 provided a higher sensitivity of 83.0% (*P*=0.043), a specificity of 82.6%, and a NPV of 82.1% in screening S-NS.

### Parallel testing algorithm of serum RPR and serum TPPA for screening S-NS

We combined the serum RPR test with the serum TPPA test in a parallel testing format, which referred to either serum RPR titer ≥1:4 or serum TPPA titer ≥1:2560, or both. With this algorithm, a significantly higher sensitivity of 87.8% (*P*<0.05) was obtained for discovering S-NS. The NPV was also raised to 84.9% (Table 3). To our surprise, using this format, the sensitivity and NPV in screening confirmed neurosyphilis could be as high as 97.5% and 98.0%, respectively. To achieve better screening performance, we set thresholds by combining serum RPR titers decreased from 1:4 to 1:2 with serum TPPA titers decreased from 1:2560 to 1:1280 (Table 3). A parallel testing format of serum RPR titer ≥1:2 and serum TPPA titer ≥1:1280 was found to be optimal, with a sensitivity of 95.8% and an NPV of 91.2% in screening neurosyphilis. Moreover, all of the confirmed neurosyphilis cases would be screened out at this threshold.

**Table 3:** Screening performance of parallel testing of serum RPR and serum TPPA for S-NS

Parallel testing format	Identification of S-NS			Identification of confirmed neurosyphilis			Identification of probable neurosyphilis		
	Sensitivity (%)	Specificity (%)	NPV (%)	Sensitivity (%)	Specificity (%)	NPV (%)	Sensitivity (%)	Specificity (%)	NPV (%)
RPR≥1:4, TPPA≥1:2560^a^	87.8	72.5	84.9	97.5	60.8	98.0	70.1	72.5	86.6
RPR≥1:2, TPPA≥1:2560	92.6	55.6	87.6	99.2	45.7	99.1	80.6	55.6	88.4
RPR≥1:4, TPPA≥1:1280	93.1	58.7	89.0	99.2	47.6	99.2	82.1	58.7	89.7
RPR≥1:2, TPPA≥1:1280	95.8	46.4	91.2	100.0	37.0	100.0	88.1	46.4	91.2

*S-NS* symptomatic neurosyphilis, *N-NS* syphilis/non-neurosyphilis, *RPR* rapid plasma reagin, *TPPA T. pallidum* particle agglutination, *PPV* positive predictive value, *NPV* negative predictive value

^a^A positive screening result was defined as either serum RPR titer ≥1:4 or serum TPPA titer ≥1:2560, or both. And by this analogy

### Univariate and multivariate analyses of factors predictive of S-NS

In the univariate analysis, previous psychiatric disorders, elevated CK and elevated homocysteine were more common in S-NS than in N-NS (*P*<0.05). Compared with N-NS, the proportion of previous ischemic stroke in probable neurosyphilis and the proportion of elevated fasting plasma glucose in confirmed neurosyphilis were higher (*P*<0.05). Notably, five S-NS patients with chronic alcoholism were all confirmed cases. There was no significant difference between S-NS and N-NS in other central nervous system infections, brain injury, brain tumors, previous hemorrhagic stroke, hypertension, heart diseases, diabetes mellitus, blood lipid, liver function or renal function (Table 4). Further multivariate analysis showed that S-NS was independently associated with male sex (odds ratio [OR], 2.376; 95% confidence interval [CI], 1.116 to 5.059), serum RPR titer ≥1:4 (OR, 3.610; 95% CI, 1.674 to 7.783), serum TPPA titer ≥1:2560 (OR, 7.290; 95% CI, 3.384 to 15.707), and elevated serum CK (OR, 4.335; 95% CI, 1.213 to 15.493). Separate analysis of confirmed and probable neurosyphilis yielded similar results (data not shown).

**Table 4:** Comparison between S-NS and N-NS in comorbidities and serum biochemical parameters

	S-NS (n=191) n (%)	N-NS (n=179) n (%)	*P* ^*a*^	S-NS		*P* ^*b*^	*P* ^*c*^
				Confirmed (*n*=123) n (%)	Probable (*n*=68) n(%)		
Comorbidities							
Other CNS infections^d^	3 (1.6)	0	0.249	3 (2.4)	0	0.067	n.a.
Brain injury	7 (3.7)	5 (2.8)	0.636	6 (4.9)	1 (1.5)	0.364	1.0
Brain tumors	2 (1.0)	3 (1.7)	0.676	1 (0.8)	1 (1.5)	0.648	1.0
Psychiatric disorders	12 (6.3)	2 (1.1)	**0.009**	7 (5.7)	5 (7.4)	**0.034**	**0.018**
History of ischemic stroke	36 (18.8)	24 (13.4)	0.156	19 (15.4)	17 (25.0)	0.618	**0.029**
History of hemorrhagic stroke	0	4 (2.2)	0.054	0	0	0.148	0.578
Hypertension	83 (43.5)	85 (47.5)	0.436	47 (38.2)	36 (52.9)	0.110	0.444
Heart diseases^e^	32 (16.8)	33 (18.4)	0.671	19 (15.4)	13 (19.1)	0.499	0.902
Diabetes mellitus	59 (30.9)	40 (22.3)	0.064	37 (30.1)	22 (32.4)	0.130	0.105
Chronic alcoholism	5 (2.6)	1 (0.6)	0.216	5 (4.1)	0	**0.043**	1.0
Serum biochemical parameters							
Elevated FPG (≥7.0 mmol/L)	38 (19.9)	24 (13.4)	0.095	30 (24.4)	8 (11.8)	**0.014**	0.731
Elevated HbA1c (≥6.5%)	36 (28.3)	29 (25.4)	0.612	22 (27.2)	14 (30.4)	0.787	0.519
Elevated CK^f^(male ≥308, female ≥192 U/L)	30 (16.9)	11 (6.6)	**0.003**	19 (17.0)	11 (16.9)	**0.006**	**0.017**
Elevated HCY^g^	42 (32.1)	22 (18.3)	**0.013**	25 (32.9)	17 (30.9)	**0.020**	0.063
Elevated TG (≥1.70 mmol/L)	41 (22.3)	41 (24.6)	0.616	22 (18.6)	19 (28.8)	0.237	0.505
Elevated CHOL (≥6.22 mmol/L)	8 (4.3)	11 (6.6)	0.355	3 (2.5)	5 (7.6)	0.120	0.778
Elevated ALT (male ≥50, female ≥40 U/L)	14 (7.3)	16 (9.0)	0.560	11 (8.9)	3 (4.4)	0.989	0.229
Elevated AST (male ≥40, female ≥35 U/L)	21 (11.1)	15 (8.5)	0.407	16 (13.0)	5 (7.5)	0.205	0.797
Elevated TBIL (≥17.1 μmol/L)	34 (18.1)	26 (15.0)	0.436	23 (18.9)	11 (16.7)	0.385	0.754
Elevated BUN (≥8.2 mmol/L)	8 (4.2)	12 (6.7)	0.285	6 (4.9)	2 (2.9)	0.510	0.361
Elevated sCr (male ≥115, female ≥97 μmol/L)	6 (3.1)	12 (6.7)	0.111	2 (1.6)	4 (5.9)	0.039	1.0

*S-NS* symptomatic neurosyphilis, *N-NS* syphilis/non-neurosyphilis, *CNS* central nervous system**,** *FPG* fasting plasma glucose, *HbA1c* glycated hemoglobin (A1c), *CK* creatine kinase, *HCY* homocysteine, *TG* triglyceride, *CHOL* cholesterol, *ALT* alanine transaminase, *AST* aspartate transaminase, *TBIL* total bilirubin, *BUN* blood urea nitrogen, *sCr* serum creatinine

*P* ^*a*^ Comparison between the S-NS group and the N-NS group, *P* ^*b*^ Comparison between the confirmed neurosyphilis subgroup and the N-NS group, *P* ^*c*^ Comparison between the probable neurosyphilis subgroup and the N-NS group

^d^Two patients had viral encephalitis and one patient had viral meningitis before being diagnosed with S-NS

^e^Heart diseases includes coronary heart disease, hypertensive heart disease, valvular heart disease and cardiac arrhythmia

^f^One patient with acute myocardial infarction was excluded

^g^Homocysteine ≥15 μmol/L in male or ≥12 μmol/L in female in <60 age group, or ≥20 μmol/L in ≥60 age group

### Combined effects of the independent risk factors of S-NS

We further analyzed the combined effects of the independent risk factors mentioned above for S-NS (Table 5). Among female syphilis patients, patients with both serum RPR titer ≥1:4, TPPA titer ≥1:2560, and normal serum CK were in greater danger than those with serum RPR titer <1:4, serum TPPA titer <1:2560 and normal serum CK, with an OR of 20.583 (95% CI, 5.853 to 72.380) for S-NS and an OR of 104.000 (95% CI, 11.575 to 934.402) for confirmed neurosyphilis. Male patients were at higher risk than female patients. Compared with female patients with serum RPR titer <1:4, serum TPPA titer <1:2560 and normal serum CK, male patients with serum RPR titer ≥1:4, serum TPPA titer ≥1:2560, and normal serum CK had a markedly increased likelihood of S-NS (OR, 53.300; 95% CI, 18.073 to 157.194), as well as confirmed neurosyphilis (OR, 265.200; 95% CI, 32.704 to 2150.534) and probable neurosyphilis (OR, 10.920; 95% CI, 3.176 to 37.549). Notably, in both male and female patients, there were 22 patients with concurrent serum RPR titer ≥1:4, serum TPPA titer ≥1:2560 and elevated CK who all had neurosyphilis (data not shown).

**Table 5:** Odds ratios of S-NS (with 95% confidence intervals) according to gender, syphilitic serological characteristics and serum creatine kinase levels

	Serum RPR titer <1:4				Serum RPR titer ≥1:4			
	Serum TPPA titer <1:2560		Serum TPPA titer ≥1:2560		Serum TPPA titer <1:2560		Serum TPPA titer ≥1:2560	
	Normal CK	Elevated CK	Normal CK	Elevated CK	Normal CK	Elevated CK	Normal CK	Elevated CK
S-NS								
Female	1.0 (Ref.)	n.a.^a^	**6.500** ^*^ **(1.273-33.201)**	n.a.^b^	2.437 (0.502-11.845)	n.a.^c^	**20.583** ^*^ **(5.853-72.380)**	n.a.^d^
Male	1.264 (0.450-3.550)	3.900 (0.734-20.709)	**9.286** ^*^ **(2.549-33.832)**	**9.750** ^*^ **(1.340-70.966)**	**4.643** ^*^ **(1.107-19.475)**	6.500 (0.357-118.370)	**53.300** ^*^ **(18.073-157.194)**	n.a.^d^
Confirmed neurosyphilis								
Female	1.0 (Ref.)	n.a.^a^	9.750 (0.507-187.526)	n.a.^b^	4.875 (0.275-86.351)	n.a.^c^	**104.000** ^*^ **(11.575-934.402)**	n.a.^d^
Male	1.083 (0.095-12.329)	n.a.^e^	n.a.^e^	**39.000** ^*^ **(2.397-634.654)**	11.143 (0.886-140.119)	n.a.^e^	**265.200** ^*^ **(32.704-2150.534)**	n.a.^d^
Probable neurosyphilis								
Female	1.0 (Ref.)	n.a.^a^	**5.850** ^*^ **(1.004-34.100)**	n.a.^b^	1.950 (0.320-11.888)	n.a.^c^	3.900 (0.734-20.709)	n.a.^d^
Male	1.300 (0.427-3.959)	4.680 (0.849-25.811)	**11.143** ^*^ **(2.913-42.620)**	3.900 (0.297-51.196)	3.343 (0.647-17.267)	7.800 (0.419-145.200)	**10.920** ^*^ **(3.176-37.549)**	n.a.^d^

*S-NS* symptomatic neurosyphilis, *N-NS* syphilis/non-neurosyphilis, *RPR* rapid plasma reagin, *TPPA T. pallidum* particle agglutination, *CK* creatine kinase, *OR* odds ratio, *Ref.* reference, *n.a.* not applicable

^*^ *P*<0.05

^a^No female patient with serum RPR titer <1:4, TPPA titer <1:2560 and elevated CK was in the S-NS group; the OR of S-NS was not applicable

^b^No female patient with serum RPR titer <1:4, TPPA titer ≥1:2560 and elevated CK was in the N-NS group; the OR of S-NS was not applicable

^c^No female patient with serum RPR titer ≥1:4, TPPA titer <1:2560 and elevated CK was in both groups; the OR of S-NS was not applicable

^d^In both male and female patients, there was no one with serum RPR titer ≥1:4, TPPA titer ≥1:2560 and elevated CK in the N-NS group; the OR of S-NS was not applicable

^e^No male patient with serum RPR titer <1:4, TPPA titer <1:2560 and elevated CK, or serum RPR titer <1:4, TPPA titer ≥1:2560 and normal CK, or serum RPR titer ≥1:4, TPPA titer <1:2560 and elevated CK was in the confirmed neurosyphilis subgroup; the OR of confirmed neurosyphilis was not applicable

## Discussion

Whether and when to conduct the CSF examination in syphilis patients continues to plague clinicians. Some risk factors of neurosyphilis have been reported in HIV-infected individuals, including male sex, higher serum RPR titers and the occurrence of neurological symptoms, as well as profiles of HIV infection (e.g., higher HIV viral load and lower peripheral blood CD4^+^ T cells) [4, 8, 10]. However, HIV-negative syphilis patients had different characteristics from HIV-infected patients [5], and predictors of neurosyphilis in HIV-negative patients have rarely been discussed, especially in S-NS. To the best of our knowledge, this is the first report to discuss this issue, and it includes a large amount of information as well as a great number of subjects. Except for male sex and increased serum RPR titers, we also found that increased serum TPPA titers and elevated serum CK can be predictors of S-NS. Moreover, patients with both serum RPR titer <1:2 and serum TPPA titer <1:1280 were unlikely to have S-NS.

Considering the invasiveness of lumbar puncture, the identification of predictors of neurosyphilis to avoid unnecessary lumbar puncture has been a hotspot in clinical research. In the present study, as the serum RPR titers gradually increased, we observed a rising trend in the likelihood of S-NS in HIV-negative patients, which coincided with a previous study of HIV-infected neurosyphilis patients [15]. In syphilis patients co-infected with HIV, serum RPR titers of 1:32 or 1:16 were recommended as indicators to determine the need for lumbar punctures in patients without neurological symptoms [9, 15]. However, among our HIV-negative subjects with neurological symptoms, a titer of 1:4 was found to be the optimal cut-off point to recognize S-NS, with a sensitivity of 76.6%. If serum RPR titer ≥1:32 or titer ≥1:16 was used to screen for S-NS, 102 (54.3%) or 78 (41.5%) cases of S-NS would be missed, respectively. Of course, if serum RPR titer ≥1:4 was used as an indicator for lumbar puncture, there were still 44 (23.4%) cases that would be missed (including seven confirmed and 37 probable neurosyphilis cases). Therefore, new indicators are urgently needed.

Conventionally, treponemal tests are considered to be irrelevant to disease activity [2]; therefore, there is little research linking the treponemal antibody with neurosyphilis. Interestingly, we found a correlation between serum RPR and serum TPPA reactivity. Furthermore, compared with serum RPR titers, serum TPPA titers even had better screening performance, with an AUC of 0.897 for identification of S-NS. Of course, the sole use of serum RPR or TPPA titer might not adequately predict S-NS in HIV-negative patients. Therefore, we conducted a parallel testing format (either serum RPR titer ≥1:4 or serum TPPA titer ≥1:2560, or both) and found that it had a higher sensitivity (87.8%) in screening S-NS than using only serum RPR or TPPA alone. To achieve a higher sensitivity, we found that the parallel testing format of serum RPR ≥1:2 and serum TPPA ≥1:1280 could screen out 95.8% of S-NS and even all confirmed neurosyphilis cases. Therefore, there is a low-probability of a missed diagnosis of S-NS in patients with both serum RPR titer <1:2 and serum TPPA titer <1:1280. The threshold also provided an NPV of 91.2%, i.e., 91.2% of patients excluded from the screening test will be correctly identified as N-NS. This parallel testing algorithm based on serological results may provide a preliminary judgment of S-NS for clinicians, especially in under-resourced settings where the lumbar puncture could not be performed in time, as well as allow for the rational use of medical resources.

The multivariate analyses showed that male sex, serum RPR titer ≥1:4, serum TPPA titer ≥1:2560, and elevated serum CK were independently associated with S-NS. Upon further analysis, the effects of these variables on the risk of developing S-NS appeared to be synergistic. Having both serum RPR titer ≥1:4 and TPPA titer ≥1:2560 increased the likelihood of S-NS with an odds ratio of 20.583 for female patients and up to 53.300 for male patients. The ORs of confirmed neurosyphilis in such patients were dramatically increased to 104.000 for female patients and up to 265.200 for male patients. As previously reported, male patients were more likely to develop neurosyphilis [6, 8]. Notably, those with serum RPR titer ≥1:4, TPPA titer ≥1:2560 and elevated serum CK were only observed in S-NS patients.

Previous studies on neurosyphilis were confined to demographic characteristics, stage of syphilis, serum RPR titer and profiles of HIV infection [4, 6, 9]. In this study, we took the comorbidities and biochemical parameters into consideration and found that serum CK could aid in the recognition of both confirmed and probable neurosyphilis. In humans, CK is found to be most concentrated and active in skeletal muscle and is released into circulation when these tissues are damaged [16]. Neurosyphilis can result in various movement disorders [17], which indicate involvement of the musculoskeletal system. In the present study, S-NS patients presented with hemiparesis or hemiplegia, dysbasia, seizure and numbness.

Our study benefited from a great number of neurosyphilis cases and variables. However, the present study is hospital-based, and only 10.4% (432/4152) of syphilis patients received lumbar puncture. Additionally, the epidemic of neurosyphilis among syphilis patients without CSF assessment was unknown. In addition, the RPR test was recommended in the CSF assessment in our country or in Europe, but the VDRL test was routinely used in the CSF assessment in previous studies, especially in the United States. Therefore, our findings may not be similar to those studies using the VDRL test. Identifying predictors of asymptomatic neurosyphilis requires further research.

## Conclusion

To effectively identify neurosyphilis cases and perform practical lumbar punctures among HIV-negative syphilis patients are real challenges, especially in neurologically symptomatic patients. Our study found that the quantitation of serum TPPA is worthwhile, and it even performs better than serum RPR in screening S-NS. Serum RPR titer ≥1:4 and serum TPPA titer ≥1:2560 were the optimal cut-off points to distinguish S-NS from N-NS. Serum RPR and serum TPPA characteristics, male sex, and serum CK can be used as auxiliary indicators of S-NS. Notably, patients with both serum RPR titer <1:2 and serum TPPA titer <1:1280 had a low probability of neurosyphilis, suggesting that it is reasonable to reduce lumbar puncture in such individuals.

## Abbreviations

AUC: Area under the ROC curve
CK: Creatine kinase
CSF: Cerebrospinal fluid
HIV: Human immunodeficiency virus
N-NS: Syphilis/non-neurosyphilis
ROC: Receiver operating characteristic
RPR: Rapid plasma reagin
S-NS: Symptomatic neurosyphilis
TPPA: *Treponema pallidum* particle agglutination
VDRL: Venereal disease research laboratory
