# Erratum to: Novel predictors of neurosyphilis among HIV-negative syphilis patients with neurological symptoms: an observational study

**DOI:** 10.1186/s12879-017-2453-2

**Published:** 2017-05-22

**Authors:** Yao Xiao, Man-Li Tong, Li-Li Liu, Li-Rong Lin, Mei-Jun Chen, Hui-Lin Zhang, Wei-Hong Zheng, Shu-Lian Li, Hui-Ling Lin, Zhi-Feng Lin, Hui-Qin Xing, Jian-Jun Niu, Tian-Ci Yang

**Affiliations:** 1 Zhongshan Hospital, Medical College of Xiamen University, Xiamen, 361004 China; 2 Xiamen Hospital of Traditional Chinese Medicine, Xiamen, 361009 China; 3 Xiamen Zhongshan Hospital, Fujian Medical University, Xiamen, 361004 China; 4 Xiamen Huli District Maternity and Child Care Hospital, Xiamen, 361009 China; 5 Institute of Neuroscience, Medical College of Xiamen University, Xiamen, 361005 China

## Erratum

After the publication of this work [1] it was noticed that the third row of Table 2 was incorrectly included as a main heading instead of being included in the body. The original article has been corrected.
